# Colorimetric loop-mediated isothermal amplification assay for detection and ecological monitoring of *Sarocladium oryzae*, an important seed-borne pathogen of rice

**DOI:** 10.3389/fpls.2022.936766

**Published:** 2022-08-18

**Authors:** Prassan Choudhary, Sanjay Kumar Goswami, Hillol Chakdar, Shaloo Verma, Shobit Thapa, Alok Kumar Srivastava, Anil Kumar Saxena

**Affiliations:** ^1^Microbial Technology Unit II, ICAR-National Bureau of Agriculturally Important Microorganisms, Mau, India; ^2^Indian Council of Agricultural Research (ICAR)-Indian Institute of Sugarcane Research, Lucknow, India

**Keywords:** *Sarocladium oryzae*, sheath rot, weeds, isothermal amplification, bio-surveillance colorimetric LAMP assay for the detection of *Sarocladium oryzae*

## Abstract

Accurate and timely disease detection plays a critical role in achieving sustainable crop protection. Globally, rice has been a staple crop for centuries plagued by the diseases that greatly hamper its productivity. Sheath rot, an emerging disease of rice caused by the seed-borne pathogen *Sarocladium oryzae*, has reportedly caused heavy losses to agricultural produce in recent years. Our study has led to the development and validation of a LAMP assay for early detection of *S. oryzae*, the causal agent of sheath rot from the live-infected tissues, seeds, weeds, and environmental samples. The assay could detect as low as 1.6 fg/μl of the pathogen in 15 min. The assay was implemented to bio-surveil the presence of this pathogen by testing it on three weed species (*Echinochloa colona*, *Echinochloa crus-galli*, and *Cyperus teneriffae*) growing around the rice fields. The results showed the presence of the pathogen in two of the weed species *viz*. *E. colona* and *E. crus-galli*. The assay was used to test 13 different rice varieties for the presence of *S. oryzae* in seeds. In total, three of the varieties did not show the presence of *S. oryzae* in their seeds while the rest were found to harbor the pathogen. The developed assay can effectively be used to detect and screen the presence of *S. oryzae* in live samples including seeds and field soil.

## Introduction

Rice sheath rot is a devastating disease complex with *Sarocladium oryzae* (Sawada) W. Gams and D. Hawkshaw as one of the main pathogens (Mvuyekure et al., 2018). Agriculture practices such as enhanced use of fertilizers, high plant density, and use of photoperiod-insensitive cultivars led to the conversion of this earlier minor disease into a major one (Mew et al., 2004; Bigirimana et al., 2015). International exchange of plant materials around the world may also have expanded the rice-*Sarocladium* pathosystem around the world (Lanoiselet et al., 2012; Bigirimana et al., 2015). Reports suggest that sheath rot can cause upto 85% yield loss in rice which can severely affect the farmers and rice cultivating nations worldwide (Sakthivel, 2001). In general, *S. oryzae* develops conidia that germinate and penetrate the plant through the stomata and wounds. At severe stages of the disease, the young panicles rising from the upper part of the rotted sheaths get affected leading to chaffiness and sterility (Prabhukarthikeyan et al., 2020; Weeraratne et al., 2020). *S. oryzae* not only affects rice but also hampers the production of crops such as sorghum, maize, and millets (Deka and Phookan, 1992; Pearce et al., 2001). Sheath rot of rice is a seed-borne disease but also survives in the infected the plant’s residues and weeds (Balakrishnan and Nair, 1981). The pathogen has also been reported to survive in soil (Bigirimana et al., 2015).

In Hittalmani et al. (2016) carried out the whole-genome sequencing of highly virulent *S. oryzae* Saro-13 which revealed the presence of unique helvolic acid and cerulenin biosynthesis pathway genes in this pathogen (Mahesh et al., 2016). Production of these secondary metabolites has a synergistic mechanism to attack the host by altering the cell permeability leading to the outflow of electrolytes in the host tissue (Sakthivel et al., 2002). In total, five major mycotoxins have been reported to contribute to the formation of rot disease. Interestingly, a recent report analyzed the pathobiomes of this disease and revealed that *Pseudomonas fuscovaginae* and *S. oryzae* are independently involved in the disease development (Musonerimana et al., 2020). The complexity of the disease along with the independent involvement of the pathogens makes its diagnosis and early detection a daunting task.

Loop-mediated isothermal amplification (LAMP) technique has revolutionized the area of pathogen diagnosis of plant diseases (Becherer et al., 2020). LAMP-based diagnostic assays have been developed for other fungal pathogens of rice-causing sheath blight (Choudhary et al., 2020). Only recently, Prasannakumar et al. (2021) reported a LAMP-based detection of *S. oryzae* and *Magnaporthe oryzae* from the infected rice seeds using the RNA Polymerase II gene. However, the study had certain disadvantages as it reported pathogen detection from infected seeds only and against a few outgroups involved in the disease complex leaving out important pathogens such as *Fusarium fujikoroi* complex (Peeters et al., 2021). The study did not take into account weeds surrounding the rice fields which act as alternate hosts all year round (Deka and Phookan, 1992; Yadav and Thrimurty, 2006).

Under this background, the present study was aimed to detect *S. oryzae* from infected sheaths as well as seeds and environmental samples such as soil with a higher sensitivity and rapidity. The alternate hosts (weeds such as *Echinochloa colona*, *E. crus-galli*, and *Cyperus teneriffae*) were also subjected to validation in this study to monitor their role as alternate hosts for the pathogen.

## Materials and methods

### Sampling and procurement of cultures and rice varieties

Cultures of *S. oryzae* with reference cultures were obtained from the National Agriculturally Important Microbial Culture Collection (NAIMCC), Mau, Uttar Pradesh, India (Table 1). Live tissue samples of sheath rot (rice and weed samples) were collected from the rice fields of ICAR–Indian Institute of Seed Science (IISS), Mau, Uttar Pradesh (Table 2). Tissue samples were collected in polypropylene bags and stored at 4°C prior to use. Seed samples of thirteen different rice varieties were generously provided Dr. Vishal Tyagi (ICAR–IISS, Mau).

**TABLE 1:** Various bacterial and fungal cultures used for LAMP assay validation.

S. No	Cultures	Obtained from
1	*Sarocladium oryzae* isolate CABI 239872	NAIMCC-F-01630
2	*S. oryzae* isolate CBS 399.73	NAIMCC-F-01631
3	*S. oryzae* isolate CABI 334318	NAIMCC-F-01632
4	*S. oryzae* isolate CABI 334320	NAIMCC-F-01633
5	*S. oryzae* isolate CABI 339944	NAIMCC-F-01634
6	*S. implicatum* RPF 22	NAIMCC-F-04128
7	*Acremonium curvulum* CABI-297016	NAIMCC-F-00022
8	*S. kiliense* ATCC-14491/CABI-090242	NAIMCC-F-00028
9	*S. strictum* ATCC-18941/CABI-230422	NAIMCC-F-00053
10	*Rhizoctonia solani* AG-1 IA isolate PU RS1	NAIMCC-F-03220
11	*R. solani* AG-1 IB isolate M2	Dr. SK Goswami, ICAR-Indian Institute of Sugarcane Research, Lucknow, India
12	*R. solani* AG 2-2IIIB isolate O1	Dr. SK Goswami, ICAR-Indian Institute of Sugarcane Research, Lucknow, India
13	*R. solani* AG-8 isolate S1	Dr. SK Goswami, ICAR-Indian Institute of Sugarcane Research, Lucknow, India
14	Magnaporthe oryzae isolate MG1	Dr. N. Sahana, UBKV, West Bengal, India
15	*R. oryzae-sativae* isolate MV1	MTCC-9666
16	*Fusarium fujikuroi* isolate RPF19	NAIMCC-F-03979
17	*Sclerotinia sclerotiorum* isolate AS1	NAIMCC-F-03341
18	*Trichoderma asperellum* isolate P2	NAIMCC-F-03330
19	*F. oxyporum* f. sp. *lycopersici*	NAIMCC-F-00889
20	*Alternaria alternata* isolate CABI-359781	NAIMCC-F-00067
21	*Ustilaginoidea virens* isolate UV2	NAIMCC-F-02995
22	*Helminthosporium oryzae* isolate 1	NAIMCC-F-03040
23	*Pseudomonas plecoglossicida* isolate S7	NAIMCC-B-00397

**TABLE 2:** Primer sequences designed for the validation of LAMP assay.

Primer name	Sequences (5′–3′)	Type	Length	GC%	T_m_ °C
SaO_act_F3	TACGCCTCTGGTCGTACC	Forward outer	18	61	61.4
SaO_act_B3	CTCCTTGATGTCACGAACGA	Backward outer	20	50	64
SaO_act_FIP	GACACGAGCAATGGCGTGGGTTTTTT-GGACTCTGGTGATGGTGT	Forward inner	21–18	61.9–55.6	73.6–58.3
SaO_act_BIP	GACATGGCTGGCCGTGATCTTTTT-GTGGTGGAGAAGGTGTAACC	Backward inner	19–20	63.2–55	69.3–60.9
SaO_act_LF	AGATGGGGACAACGTGAGTG	Forward loop forming	20	55	65.1
SaO_act_LR	ATTACCTCATGAAGATCCTTGCTGA	Backward loop forming	25	40	65.7

### Template preparation from live sheath samples and DNA extraction from seeds

A paper punch was used to cut out two leaf discs (∼3 mg) from the diseased and healthy sheaths and added to a 0.2 ml PCR tube. In total, 30 μl of elution buffer (HiMedia Laboratories Pvt., Ltd., Mumbai, India) was added to the tube and, the discs were macerated using a sterile 100 μl tip for 2 min for lysis. From the lysate, 3 μl was used for the LAMP assays. DNA extraction from rice seeds was carried out following the method described by Júnior et al. (2016). In brief, the seeds (200 mg) were ground in 1 ml of pre-heated (65°C) extraction buffer containing CTAB (2% w/v), Tris–HCl (100.0 mM, pH 8.0), EDTA (20.0 mM, pH 8.0), NaCl (1.4 M), PVP (1.0% w/v), and β-mercaptoethanol (3 mM). Then, the ground material was transferred to a microcentrifuge tube (2.0 ml) and homogenized carefully. After that the tubes were incubated at 65°C for 1 h and inverted after every 15 min. At the end of incubation, Chloroform: Isoamyl alcohol (96:4) was added and vortexed for 1 min. The tubes were centrifuged 5,000 *g* for 10 min at 4°C and supernatant was collected. To this supernatant, three volumes of a solution (pH 5.2) containing ethanol (86% v/v) and ammonium acetate (1.07 M) was added and incubated at −20°C for 1 h. The entire content was centrifuged at 10,000 rpm for 1 min and pellets were collected which were then suspended to 100 μl TE buffer. To these three volumes of a solution (pH 4.7) containing ethanol (95% v/v) and sodium acetate (143.0 mM) was added and at −20°C for 1 h. After incubation, pellets containing DNA were obtained by centrifugation (10,000 rpm) and resuspended in 80 μl TE buffer.

### Loop-mediated isothermal amplification assay validation, specificity, and sensitivity

#### Primer designing

Primers for the LAMP assay were designed using the partial actin gene (NCBI accession number : HG964979.1) from *S. oryzae* strain CBS 180.74. Primer Explorer v5 was used to design six primer sets (Eiken Chemical Co., Ltd., Tokyo, Japan). The detailed properties of the primers have been mentioned in Table 2. All the primers were synthesized by Eurofins Genomics India Pvt. Ltd., India. Primer BLAST tool from NCBI was used to check the specificity of the designed primers.

#### Loop-mediated isothermal amplification assay conditions and gel electrophoresis

The LAMP system used in this study consisted of 12.5 μl WarmStart Colorimetric LAMP 2X Master Mix (New England Biolabs Inc., Ipswich, MA, United States), 2.5 μl primer mix (SaO_act_F3/SaO_act_B3, SaO_act_FIP/SaO_act_BIP, and SaO_act_LF/SaO_act_LR) (here, we assign the primer sets as SaO LAMP primers), 3 μl template DNA, and Mili-Q water (Promega, New Delhi, India) for a final volume of 25 μl. The assay conditions were followed according to the manufacturer’s protocol (65°C for 30 min). The assay was also tested keeping the assay time at 15 min. Visual confirmation was carried out as yellow color development indicated positive reaction while red/pink color indicated no reaction. The amplified LAMP products were further observed on 2% agarose gel with ethidium bromide staining to confirm the amplifications. In total, 5 μl of each amplification product was loaded on agarose gel and was run under 55 V for 2 h.

#### Sensitivity of loop-mediated isothermal amplification assay

The sensitivity of the LAMP assay was determined using different *S. oryzae* DNA concentrations in descending order by 10-fold serial dilution with sterilized double-distilled water from 160.4 ng/μl to 1.604 × 10^–2^ fg/μl. Serially diluted DNA (3 μl each) was used as template DNA in the LAMP reaction to quantify its sensitivity in a thermal cycler at a uniform temperature of 65°C for 30 min.

#### Specificity of loop-mediated isothermal amplification assay

To check the specificity, fungal and bacterial cultures listed in Table 1 were used. A “no template control (NTC)” was kept in all the experimental set ups and the experiments were repeated three times.

### Validation of loop-mediated isothermal amplification assay using soil DNA

In order to check the applicability of the assay, soil samples were collected from rice fields at ICAR-IISS, Mau, India. Samples were collected according to the procedure described earlier (Choudhary et al., 2020). Total soil DNA was extracted from the pooled sample in six replications using a commercial kit (FastDNA Spin Kit for Soil, MP Biomedicals, Santa Ana, CA, United States) following minor modifications (1 h for incubation period with DNA-binding buffer) to the manufacturer’s protocol. DNA was quantified using NanoDrop (Thermo Fisher Scientific, India) and pooled DNA sample was used for the LAMP assay. The time for LAMP assay was optimized and the final assay conditions were 65°C for 45 min with deactivation at 80°C for 5 min.

### Validation on weed species and different varieties of rice seeds

The diseased sheath of the weeds was cut and lysed following the rapid high throughput template preparation (rHTTP) protocol (Choudhary et al., 2019). Templates were used for carrying out the LAMP assay keeping the optimized conditions as described earlier. In case of seeds, 2 μl the total genomic DNA was used for the assay keeping all the other conditions constant.

### Artificial infection of rice with *Sarocladium oryzae*

Pure culture of *S. oryzae* NAIMCC-01633 was inoculated in PDA plates for 4 days until a uniform fungal mat developed on the media surface. Rice seeds of TKM13 were sterilized with 1.0% sodium hypochlorite and placed on the fungal mat (5 seeds/plate). The seeds were collected after 24 and 48 h, washed with sterile water and proceeded for DNA extraction as described in the Methods Section “template preparation from live sheath samples and DNA extraction from seeds.”

## Results

### Validation and specificity of the loop-mediated isothermal amplification assay for *Sarocladium oryzae* detection

**FIGURE 1:**
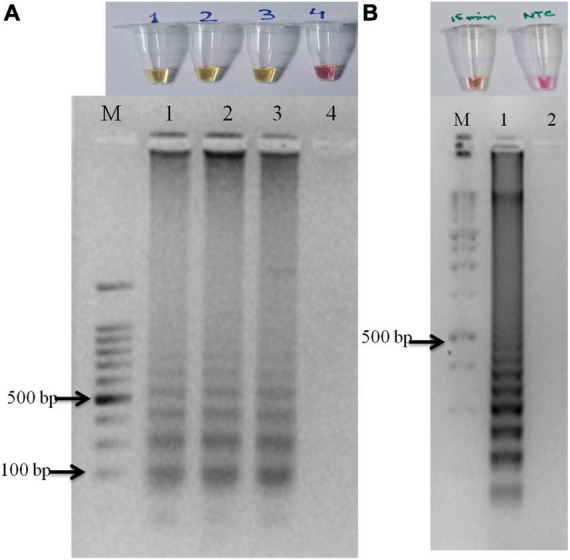
Optimization and validation of LAMP assay. Yellow color indicated a positive reaction while red/pink color indicated no reaction. **(A)** LAMP assay optimized with pure fungal isolates with no template control. **(B)** M: 100 bp (Promega); 1: *S. oryzae* NAIMCC-F-01633; 2: No Template control. The assay time was kept at 15 minutes.

**FIGURE 2:**
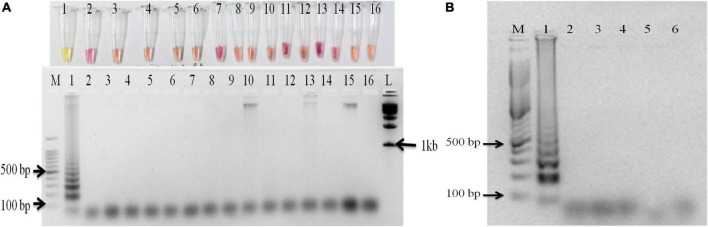
Specificity assay of the LAMP assay. **(A)** M: 100 bp (Promega); 1: *S. oryzae* NAIMCC-F-01630; 2: *Rhizoctonia solani* AG-1 IA isolate NAIMCC-F-03220; 3: *R. solani* AG-1 IB isolate M2; 4: *R. solani* AG 2-2IIIB isolate O1; 5: *R. solani* AG-8 isolate S1; 6: *Magnaporthe oryzae* isolate MG1; 7: *R. oryzae-sativae* isolate MTCC-9666; 8: *Fusarium fujikuroi* NAIMCC-F-03979; 9: *Sclerotinia sclerotiorum* NAIMCC-F-03341; 10: *Trichoderma asperellum* NAIMCC-F-03330; 11: *F. oxyporum* f. sp. *lycopersici*; 12: *Alternaria alternata* isolate NAIMCC-F-00067; 13: *Ustilaginoidea virens* NAIMCC-F-02995; 14: *Helminthosporium oryzae* NAIMCC-F-03040; 15: *Pseudomonas plecoglossicida* NAIMCC-B-00397; 16: No Template control; (upper panel: gel photograph; lower panel: colorimetric reactions in PCR tubes). **(B)** M: 100 bp (Promega); 1: *S. oryzae* NAIMCC-F-01631; 2: *S. implicatum* RPF 22 NAIMCC-F-04128; 3: *Acremonium curvulum* CABI-297016 NAIMCC-F-00022; 4: *S. kiliense* ATCC-14491/CABI-090242 (*A. kiliense*) NAIMCC-F-00028; 5: *S. strictum* ATCC-18941 CABI-230422 (*A. strictum*) NAIMCC-F-00053; and 6: No Template control.

**FIGURE 3:**
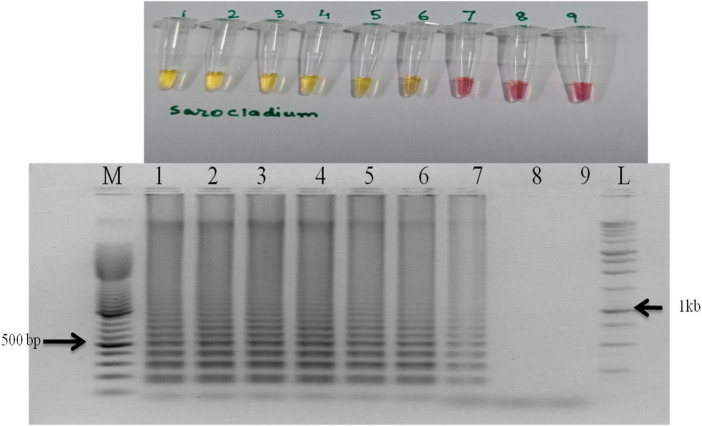
Sensitivity of the LAMP assay when performed with 10-fold serial dilution of template DNA (*S. oryzae* NAIMCC-F-01630). M: 100 bp (Promega); 1: 160.4 ng/μl; 2: 160.4 × 10^–1^ ng/μl; 3: 160.4 × 10^–2^ ng/μl; 4: 160.4 × 10^–3^ ng/μl; 5: 160.4 × 10^–4^ ng/μl; 6: 160.4 × 10^–5^ ng/μl; 7: 160.4 × 10^–6^ ng/μl; 8: 160.4 × 10^–7^ ng/μl; 9: No template control; and L: 1 kb (Promega). Upper panel in the figure shows reaction tubes whereas lower panel shows agarose gel electrophoresis results.

The primer set designed for the study targeted actin gene in *S. oryzae* strain CBS 180.74 (Ou et al., 2020). The SaO LAMP primer sets could detect *S. oryzae* successfully while there was no amplification in NTC (Figure 1A). The assay could successfully detect the pathogen in 15 min of reaction time as well (Figure 1B). In terms of specificity, the primer set specifically detected the *S. oryzae* isolates, but did not amplify in other reference fungi and bacteria tested (Figure 2A). Also, the primer set showed specific amplification in *S. oryzae* NAIMCC-01633 while no amplification was observed among other *Sarocladium* species *viz. S. implicatum* RPF 22, *S. kiliense* ATCC-14491, *S. strictum* ATCC-18941, and *Acremonium curvulum* CABI-297016 (Figure 2B). The LAMP assay did not show any amplification for other prominent rice pathogens as well. The assay could detect the target gene in as low as 1.6 fg/μl of the template DNA (Figure 3).

## Detection from live-infected and environmental samples

**FIGURE 4:**
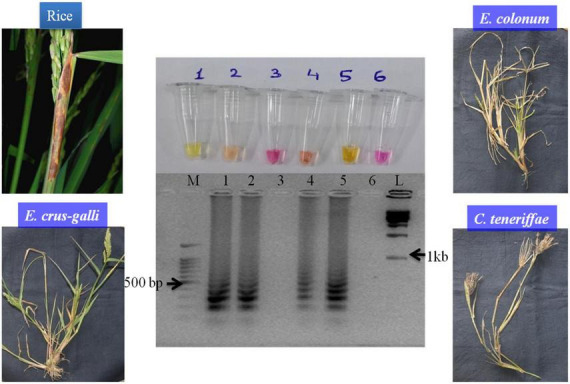
The LAMP assay with infected rice leaves along with three weed species. M: 100 bp (Promega); 1: *S. oryzae* NAIMCC-F-01632; 2: *S. oryzae* infected tissue; 3: *Cyperus teneriffae* infected tissue; 4: *Echinochloa crus-galli* infected tissue; 5: *E. colona* infected tissue; and 6: No template control; L: 1 kb (Promega). Middle panel in the figure shows agarose gel electrophoresis results, lower panel shows diseases severity in the samples and upper panel shows reaction tubes.

When diseased, live samples from rice and weeds were tested with SaO LAMP primer sets, it could detect *S. oryzae* from rice, *E. colona*, and *E. crus-galli* sheaths while no amplification was obtained in infected sheaths of *C. teneriffae* and NTC (Figure 4). The overall assay time starting from template preparation to the detection was quite less as the diseased tissues could be prepared as template in 5 min only bypassing plant genomic DNA isolation which is a costly and time-consuming task.

Furthermore, the extracted soil DNA samples were quantified (145–160 ng/μl) and the developed assay was validated. The LAMP assay gave good results when the assay time was of 45 min for the visual confirmation in the case of soil DNA as template (Figure 1). Clearly, the primer sets could detect the presence of *S. oryzae* in the soil samples obtained from the rice fields.

## Detection of *Sarocladium oryzae* in rice varieties

**FIGURE 5:**
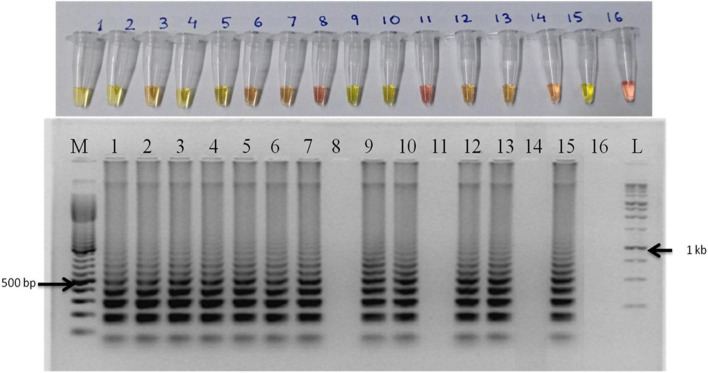
M: 100 bp (Promega); 1: artificial inoculation with *S. oryzae* NAIMCC-F-01631; 2: artificial inoculation with *S. oryzae* NAIMCC-F-01634; 3: Pusa Basmati 1121; 4: RNR 2415; 5: Rajendra sweta; 6: Swarna MTU 7024; 7: Kalanamak BK-102; 8: TKM 13; 9: Kalanamak KN 3; 10: BPT 5204; 11: Saryu 52; 12: CO- 51; 13: Kalanamak 101; 14: Swarna sub 1; 15: Rajendra kasturi; and 16: No template control.

Out of thirteen rice varieties, 10 showed the presence of the seed pathogen *S. oryzae* (Table 3). The varieties which did not show amplification were TKM13, Saryu 52, and Swarna Sub 1 (Figure 5). No amplification was observed in NTC.

**TABLE 3:** Details of rice varieties used in the study.

S. No	Varieties of rice seeds	LAMP assay validation (+/−)
01	Pusa basmati 1121	+
02	RNR 2415	+
03	Rajendra sweta	+
04	Swarna MTU 7029	+
05	Kalanamak BK-102	+
06	TKM 13	−
07	Kalanamak KN 3	+
08	BPT 5204	+
09	Saryu 52	−
10	CO – 51	+
11	Kalanamak 101	+
12	Swarna sab 1	−
13	Rajendra kasturi	+

+ denotes the presence of S. oryzae; − denotes the absence of S. oryzae.

## Discussion

Rice is one of the most prominent crops which cater to the needs of the ever-growing population of the world. With constant decline in agricultural lands and loss in production due to diseases, it is becoming increasingly difficult to meet the global food demands (Godfray et al., 2016). Losses in production due to diseases can be minimized through early detection of diseases which paves way for easy and environmental friendly management techniques. Molecular detection techniques of pathogens have advanced over the years replacing PCR-based diagnostic methods because of the high cost, non-availability of sufficient centralized laboratories besides being time-consuming. Even machine learning–based computational techniques have been developed to diagnose plant pathogens which show the importance of timely detection of diseases (Mohanty et al., 2016). LAMP-based assays have widely been reported in case of phytopathogens because of their high sensitivity, rapidity, and ease of result visualization. Recently, Choudhary et al. (2020) developed a LAMP assay for the early detection of sheath blight pathogen (*Rhizoctonia solani* AG-1 IA) in rice. Ortega et al. (2018) reported the detection of *F. fujikoroi* and *M. oryzae* from rice seeds. Recently, Jiang et al. (2021) developed a LAMP assay for detecting *Fusarium* spp. in rice seeds. LAMP assays have successfully been used in field conditions for the identification of various plant pathogens (Blaser et al., 2018; Rizzo et al., 2020). Keeping these recent reports in mind, we tested and validated a LAMP assay for timely and easy detection of *S. oryzae*, a seed-borne fungal pathogen causing sheath rot of rice.

For the detection of *S. oryzae*, three critical points were to be considered: (1) multiple pathogens associated with sheath rot (Peeters et al., 2021), (2) presence of alternate hosts around the rice fields, and (3) presence of pathogen in the seeds, tissues and soil. Therefore, any diagnostic assay should be specific and sensitive enough to detect the target pathogen form a mixture of associated microorganisms present in tissues, seeds or environmental samples. For a diagnostic marker to be useful against *S. oryzae*, it must provide early detection irrespective of the developmental stage of the pathogen (Raju et al., 2020).

Although LAMP-based methods are time saving and can be performed in field conditions, isolation of DNA from target tissues becomes a major hurdle. If the target plant tissue has polyphenolics or high number of polysaccharides, then systematic genomic DNA isolation methods require liquid nitrogen or costly commercial kits (Choudhary et al., 2019). In order to overcome these problems, we used a simple template preparation technique which is time saving (preparation took 5 min), applicable in field conditions, and compatible with the polymerase enzyme used in the LAMP assay. The assay was highly specific as it showed positive amplification for *S. oryzae* but did not show any amplification for other pathogens especially for *F. fujikuroi* which is also known to be associated with sheath rot (Bigirimana et al., 2015). Remarkably, the assay was specific to *S. oryzae* only among other *Sarocladium* spp. The high sensitivity (1.6 fg/μl) of the assay will play a major role as it can detect even small quantities of the presence of the pathogen as compared to that (100 fg/μl) for the assay developed earlier by Prasannakumar et al. (2021) for this pathogen.

Although, not many molecular detection assays are available for *S. oryzae*, but obviously, any of the PCR or qPCR-based assay will take much longer time for detection. Recently, Lin et al. (2021) reported a qPCR- and TaqMan PCR-based molecular detection of rice from field infected samples. The study emphasized the rapid detection of samples but the assays used clearly takes more than 2 h in each of the techniques used. Earlier, Cui et al. (2016) used multiplex PCR to detect six bacterial pathogens in rice. Multiplex PCR assays have the same drawback as it takes much time and require state-of-the-art laboratories. Even the LAMP-based assay developed by Prasannakumar et al. (2021) required at least 30 min for detection of *S. oryzae*.

It is important to note that there are some rice varieties such as MDR40049, CN 1035-61, BPMSl, BPM-40A, Kala Namak, KSR white, Basmati 10, Swarna HAU 11-12, PAU 269-1-9-2-1, HPU 8106, TR-B-63, and OR-090-3-158 which are resistant to sheath rot but are not very popular in the major rice-growing regions (Fetene et al., 2020; Pushpam et al., 2020). In the present study, we also tried to see if the assay can detect the pathogens in some of the available rice varieties including a few popularly grown ones. The LAMP assay developed in this study detected the presence of *S. oryzae* in seeds of all the three Kala Namak varieties (BK-102, KN 3, and 101). The pathogen could not be detected in Swarna Sub 1 which a known resistant variety toward sheath rot (Pushpam et al., 2020). Also, Saryu 52 and TKM 13 seeds confirmed the absence of *S. oryzae* that are only moderately resistant varieties (Banumathy et al., 2016). This study points out an urgent need for screening of rice varieties/lines against this pathogen to develop an exhaustive database for resistance/susceptibility. Furthermore, the seed lots distributed to the farmers for cultivation should also be routinely checked to avoid any sudden build up and epidemic of this disease.

Bio-surveillance of alternate hosts comes into play to get an idea whether the pathogen is surviving on alternate hosts in a particular area or not. Common weed plants such as *E. colona*, *Monochoria vaginalis*, *Hymenachne assamica*, *Leersia hexandra*, *Panicum walense*, *Oryza rufipogon*, and *Eleusine indica* have already been reported to be infected by *S. oryzae* (Rahman et al., 1982; Deka and Phookan, 1992). Out of three weeds growing around the rice fields in IISS, Mau, we could detect the presence of *S. oryzae* in *E. colona*, and *E. crus-galli* using the LAMP assay. These weeds growing around the rice fields provide shelter to this pathogen all year round and aid in infection of rice plants during the crop season (Balakrishnan and Nair, 1981; Yadav and Thrimurty, 2006). Similarly, the pathogen can also survive in soil and plant residues found in fields (Peeters et al., 2021). Diagnostic assays compatible with environmental samples such as soil samples from agricultural fields, would definitely give way to a robust, early and affordable disease management (Shekhawat et al., 2020; Zhang et al., 2021). The LAMP assay developed in the study was very much capable of detecting the pathogen in soil as well.

The LAMP-based diagnostic assay developed in this study was fast, highly sensitive, specific in the detection of *S. oryzae*. Furthermore, the ability of this assay to detect the pathogen in soil and weeds is definitely an edge over the other similar assays developed for the pathogen. These features along with minimal requirement of instrumentation for LAMP assays make it very suitable for using it as field scale assay. Moreover, the assay can be effectively used to screen rice seed lots at air and sea ports where the exchange of plant material takes place on a large scale. This would provide as a checkpoint on the propagation of *S. oryzae* around the globe from an ecological monitoring point of view.

## Data availability statement

The original contributions presented in this study are included in the article/supplementary material, further inquiries can be directed to the corresponding author.

## Author contributions

HC conceptualized and designed the experiments. PC and SV executed the experiments. PC with the help of SG compiled the results and prepared the first draft of the manuscript. HC and ST revised the first draft of the manuscript. HC, AlS, and AnS edited and finalized the manuscript. All authors contributed to the article and approved the submitted version.
